# Functional Heterogeneity of Canine Osteosarcoma Cell Lines and Differential Expression of *miR-27b-3p* and IGF2BP3

**DOI:** 10.3390/cells15100878

**Published:** 2026-05-12

**Authors:** Emilia Magdalena Łukasik, Klaudia Aneta Marcinkowska, Agnieszka Śmieszek

**Affiliations:** Laboratory of Preclinical Research “In VetBio”, Department of Pharmacology and Toxicology, Faculty of Veterinary Medicine, Wroclaw University of Environmental and Life Sciences, Norwida 31, 50-375 Wroclaw, Poland; agnieszka.smieszek@upwr.edu.pl

**Keywords:** bone tumor, canine osteosarcoma, comparative oncology, gene expression, cytophysiology, in vitro model

## Abstract

**Highlights:**

**What are the main findings?**

- OSCA8, OSCA29, and D17 canine osteosarcoma cell lines exhibited distinct functional phenotypes in assays evaluating metabolism, proliferation, clonogenicity, and migration.
- The analyzed cell lines differed in miR-27b-3p and IGF2BP3 expression patterns, with OSCA29 displaying a particularly distinct molecular profile at both transcript and protein levels.

**What are the implications of the main findings?**

- Cell line-specific functional and molecular profiles should be taken into account when selecting in vitro models for canine osteosarcoma research.
- Integrated phenotypic and molecular characterization may improve interpretation and reproducibility of comparative oncology research involving canine osteosarcoma models.

**Abstract:**

Canine osteosarcoma (OSA) is a highly aggressive primary bone tumor and a valuable model in comparative oncology. Nevertheless, commonly used canine in vitro models remain incompletely and inconsistently characterized, while exhibiting substantial biological heterogeneity affecting experimental outcomes. This study aimed to comparatively characterize three canine osteosarcoma cell lines (OSCA8, OSCA29, and D17) in reference to canine hTERT fibroblasts, and with a focus on functional properties and selected molecular features, namely including *miR-27b-3p* and IGF2BP3 expression. The cytophysiological profile of the cells was evaluated in relation proliferation and migratory capacity. In turn, gene expression was determined with RT-qPCR, and proteins detected with Western blotting. The D17 cell line showed the highest metabolic activity and the largest fraction of S-phase cells, whereas OSCA8 cells demonstrated the greatest clonogenic potential and the highest migratory activity in the wound healing assay. OSCA29 cells displayed an intermediate functional profile, while all OSA cell lines exhibited comparable migratory capacity in transwell assay. At the molecular level, *miR-27b-3p* expression was significantly higher in OSCA8 and D17 cells than in OSCA29 cells. In turn, *IGF2BP3* transcript levels were lower in OSCA29 cells, whereas protein analysis revealed distinct immunoreactive forms. Together, these findings highlight the functional heterogeneity of commonly used canine osteosarcoma cell lines and broaden their current characterization.

## 1. Introduction

Osteosarcoma (OSA) is a malignant bone-forming tumor of mesenchymal origin, characterized by osteoid production by neoplastic cells, aggressive local invasiveness, and a strong tendency to metastasize [1,2,3]. This neoplasm occurs in humans and dogs, with canine osteosarcoma the most common primary bone tumor in dogs [4,5]. Canine osteosarcoma occurs disproportionately often in large and giant breeds, including Rottweilers, Great Danes, Irish Wolfhounds, and Saint Bernards, suggesting a relationship with rapid bone growth and body size, and has also been linked to dysregulation of the IGF-I/GH axis [6,7]. This molecular pathway is crucial for normal bone growth, but when dysregulated, it may generate a potent pro-proliferative signal contributing to uncontrolled cell division [8]. In large-breed dogs, elevated systemic concentrations of IGF-I may exert pro-tumorigenic effects through interaction with cancer cells exhibiting aberrantly high IGF-1R expression. This interaction initiates downstream AKT and MAPK signaling, fostering cell proliferation and impairing apoptotic processes [9].

Importantly, canine osteosarcoma shares multiple clinical, histological, and molecular features with its human counterpart [4,6,7]. Because it arises spontaneously in immunocompetent animals and follows a similarly aggressive course, it constitutes a valuable model in comparative oncology and an important bridge between naturally occurring disease and translational cancer research [4,6,10].

In this context, canine osteosarcoma-derived in vitro models represent a useful platform for investigating tumor biology and evaluating therapeutic responses under controlled conditions [10,11]. At the same time, these models are not biologically uniform [10,12]. Distinct canine osteosarcoma cell lines may differ in morphology, proliferation dynamics, metabolic activity, migratory behavior, and molecular background, all of which can influence the outcome and interpretation of experimental studies [12]. Cellular metabolism, proliferative capacity, and migratory potential represent fundamental determinants of tumor aggressiveness and therefore constitute key parameters in the comparative characterization of osteosarcoma models [13,14].

In the present study, particular attention was given to three canine osteosarcoma cell lines: OSCA8, OSCA29, and D17 (Table A1). The cell lines were selected as established and experimentally tractable models with different biological backgrounds that may capture different aspects of canine osteosarcoma heterogeneity [10]. These cell lines serve as an established and widely cited model in oncological research [10,15,16]. Their application facilitates a deeper understanding of the molecular and immunological foundations of tumor biology, elucidates the complex mechanisms of metastasis, and drives the development of innovative therapeutic strategies, such as metabolic targeting and cell cycle regulation [10].

OSCA8 and OSCA29 are primary tumor-derived cell lines with spindle-like morphology [17], whereas D17, one of the most commonly used canine osteosarcoma cell lines, originates from a metastatic lesion to the lung and displays a more epithelial-like phenotype [15,18,19]. Thus, the use of these three models enables comparison between primary tumor-derived and metastatic-derived osteosarcoma cells, while also accounting for differences in morphology and presumed biological behavior. Moreover, comprehensive baseline characterization of canine osteosarcoma cell lines remains essential to support reliable model selection and interpretation in comparative oncology studies.

Among the molecular factors potentially involved in osteosarcoma progression, IGF2BP3 has attracted increasing attention [20,21,22,23]. IGF2BP3 is an RNA-binding protein that regulates mRNA stability and translation and is highly expressed during embryonic development, whereas its expression in normal adult tissues is very low or absent [21,22]. Re-expression of IGF2BP3 has been described in numerous malignancies and is generally associated with a more aggressive phenotype [21,22,24,25]. In osteosarcoma, its overexpression has been linked to enhanced tumor progression, increased invasiveness, and metastatic potential, suggesting that IGF2BP3 may serve not only as a marker of malignant behavior but also as a relevant regulator of oncogenic processes [24,26,27].

Another important layer of post-transcriptional regulation is provided by microRNAs. For example, *miR-27b-3p* has been implicated in regulating proliferation, migration, survival, and cancer-related signaling pathways [28,29,30]. However, data on *miR-27b-3p* and IGF2BP3 expression in canine osteosarcoma cell lines, particularly in relation to their phenotypic characteristics, remain limited.

Therefore, this exploratory study aimed to establish a comparative baseline profile of selected canine osteosarcoma cell lines, focusing on metabolic activity, proliferative status, clonogenicity, migration, invasion, and the expression of *miR-27b-3p* and IGF2BP3 at both the transcript and protein levels.

## 2. Materials and Methods

### 2.1. Cell Culture and Characterization

All canine cell lines used in the study were obtained from established cell culture collections. OSCA8 and OSCA29 lines were purchased from Kerafast (Newark, CA, USA), while D17 were derived from European Collection of Authenticated Cell Cultures (ECACC, Salisbury, UK) An immortalized canine fibroblast line, hTERT, derived from skin tissue, was used as a control. These cells were purchased from Applied Biological Materials (ABM, Richmond, BC, Canada). Prior to the experiments, all cell lines were expanded in T75 culture flasks (Nunc, Biokom, Janki, Poland) using the appropriate complete growth media (CGM) comprising the appropriate base medium and supplements. The OSCA8 and OSCA29 lines were maintained in Dulbecco’s Modified Eagle Medium (DMEM; Gibco, Thermo Fisher Scientific, Warsaw, Poland). For the D17 line, Eagle’s Minimum Essential Medium (EMEM; Sigma-Aldrich/Merck, Poznań, Poland) was used, while hTERT fibroblasts were cultured in PriGrow III Medium (ABM, Richmond, BC, Canada). Each basic medium was supplemented with 10% Fetal Bovine Serum (FBS; Gibco, Thermo Fisher Scientific, Warsaw, Poland) and 1% Penicillin-Streptomycin (P/S; Sigma-Aldrich/Merck, Poznań, Poland). Additionally, CGM for OSCA-8 and OSCA-29 was supplemented with 1% HEPES buffer (HEPES Buffer Solution, Gibco, Thermo Fisher Scientific, Warsaw, Poland). Standard culture conditions were maintained, including incubation in a CO2 atmosphere at a constant temperature of 37 °C and 95% humidity. Cells were passaged when confluence reached 70% to 90%.

OSCA8, OSCA29, D17, and hTERT cells at passages 13, 10, 54, and 9, respectively, were used for the experiments. The absence of Mycoplasma contamination in D-17 cells was confirmed by IDEXX BioAnalytics (Kornwestheim, Germany), whereas OSCA-8, OSCA-29, and hTERT cells were tested using the Venor^®^GeM qOneStep Mycoplasma Detection Kit (Minerva Biolabs GmbH, Berlin, Germany).

### 2.2. Evaluation of Cell Morphology with Epifluorescent Microscope

To evaluate cell morphology and growth pattern, cells were seeded onto 24-well plates, covered with sterile coverslip in each well. The cells were directly seeded onto its surface at a density of 30,000 cells per well in 0.5 mL of CGM. After 24 h of incubation in a CO_2_ atmosphere at 37 °C, the cells were fixed with 4% paraformaldehyde. In the subsequent step, cells were permeabilized with 0.2% Tween 20 solution (Sigma-Aldrich/Merck, Poznan, Poland) by a 20 min incubation. Phalloidin atto-488 (diluted 1:800 in HBSS) was used to visualize the cytoskeleton, and the staining process was conducted at 37 °C for 30 min. Cell nuclei were counterstained using 4′,6-diamidino-2-phenylindole dihydrochloride (DAPI), provided in the ProLong™ Diamond Antifade Mounting with DAPI (Thermo Fisher Scientific, Warsaw, Poland). The prepared slides were then visualized using epifluorescence microscopy (Leica DMI8, Wetzlar, Germany) at 100-fold magnification, with images captured by a Leica K3M camera (Wetzlar, Germany). The analysis employed two fluorescence channels for phalloidin (excitation at 493 nm, emission at 517 nm) and (i) DAPI (excitation at 353 nm, emission at 465 nm).

### 2.3. Evaluation of Osteosarcoma Cell Lines Proliferation and Migration

To evaluate the proliferative and migratory capacities of the studied cells, the following assays were performed: MTS, CFU, wound-healing, cell cycle analysis, and Transwell migration assay.

#### 2.3.1. MTS Assay

The MTS assay was employed to assess metabolic activity using a colorimetric reagent (MTS; Cell Proliferation, Colorimetric, Abcam, Cambridge, UK), which served as an indicator of cell proliferation. Cells were seeded directly onto 96-well plates in a density gradient ranging from 1000 to 20,000 cells per well. The cells were suspended in CGM)at a volume of 200 μL per well, including control samples. After 24 h of incubation, 20 μL of MTS reagent was added to each well. The plates were incubated in a CO_2_ incubator at 37 °C for 2 h. Subsequently, absorbance was measured at 490 nm using a spectrophotometer (Epoch Biotek, Biokom, Janki, Poland).

#### 2.3.2. Cell Cycle Analysis

Cells for cell cycle analysis were cultured in T25 flasks until reaching 60–70% confluence. The cells were washed with Hanks’ Balanced Salt Solution (HBSS), detached using trypsin (StableCell^TM^ Trypsin solution, Sigma-Aldrich/Merck, Poznan, Poland), and fixed in chilled 70% ethanol; all reagents were purchased from Sigma-Aldrich/Merck, Poznan, Poland. The prepared samples were processed using the FxCycle™ PI/RNase Staining Solution (Invitrogen Life Technologies, Warsaw, Poland). The stained cells were examined using a CytoFLEX flow cytometer (Beckman Coulter, Brea, CA, USA). The gating strategy included initial exclusion of debris based on FSC/SSC parameters, followed by singlet discrimination using FSC-A versus FSC-H and/or FSC-W. For each condition, 30,000 single-cell events were acquired. Gates were defined using appropriate controls and applied consistently across all samples. The DNA-content histograms were evaluated within the singlet population using a standard cell-cycle model. Data analysis was performed using CytExpert software (version 2.4; Beckman Coulter, CA, USA).

#### 2.3.3. Colony Forming Unit (CFU) Assay

The CFU assay was performed on 6-well plates. Each well was seeded with 2 mL of cell suspension in CGM at a density of 10 cells/cm^2^. Following the incubation period, the cells were fixed with 4% paraformaldehyde (PFA) and stained with a 2% pararosaniline solution (Sigma-Aldrich/Merck, Poznan, Poland). The resulting colonies were counted, with a colony defined as a cluster containing more than 50 cells.

#### 2.3.4. Wound Healing Assay (Scratch Test)

The wound-healing assay was performed in 6-well plates, seeded with 500,000 cells per well in 2 mL of CGM. To ensure reliability, cells were maintained at a high confluence of approximately 90%. A vertical scratch was made in each well using a 200 μL pipette tip. The cultures were then incubated for an additional 24 h to allow for cell migration. After incubation, the cells were fixed with 4% PFA and stained with a 2% pararosaniline solution (both reagents from Sigma-Aldrich/Merck, Poznan, Poland).

#### 2.3.5. Transwell Migration Assay

The migration capacity of the cell lines was evaluated using a Transwell assay on 24-well plates. Each well contained approximately 0.5 mL of CGM. Porous inserts (ThinCerts, Greiner Bio-One, Kremsmünster, Austria) were placed above, and contained cells suspended in 0.3 mL serum-free medium (FBS-free). After 48 h, the cells that migrated to the bottom surface of the inserts were fixed with 4% PFA and stained with 2% pararosaniline solution (both Sigma-Aldrich/Merck, Poznan, Poland).

### 2.4. Gene Expression Analysis

To analyze gene expression, the RT-qPCR method was employed. The first stage involved total RNA isolation from cultured cells using the phenol–chloroform extraction method with TRI Reagent (Sigma-Aldrich/Merck, Poznań, Poland), according to the manufacturer’s protocol. The isolated RNA was diluted in nuclease-free water (Sigma-Aldrich/Merck, Poznań, Poland), and its concentration and purity were assessed spectrophotometrically at 260, 280, and 230 nm using a DS-11 Fx spectrophotometer (DeNovix, Wilmington, DE, USA). RNA purity was evaluated based on both A260/A280 and A260/A230 absorbance ratios. Only RNA samples with both absorbance ratios within accepted ranges, i.e., approximately 1.8–2.1 were used for downstream analyses. Potential genomic DNA (gDNA) contamination was removed using DNase I from the PrecisionDNase kit (Primerdesign, Blirt DNA, Gdańsk, Poland).

Purified RNA (500 ng) was reverse-transcribed into cDNA using the Tetro cDNA Synthesis Kit (Bioline Reagents Limited, London, UK) for gene expression detection, while cDNA for miRNA detection was synthesized from 375 ng of RNA using Mir-X ™ miRNA First-Strand Synthesis Kit (Takara Clontech Laboratories, Biokom, Poznan, Poland) Reactions were conducted in a T100 Thermal Cycler (Bio-Rad, Hercules, CA, USA) according to the manufacturer’s instructions.

The quantitative PCR (qPCR) was performed using 1 μL of cDNA template with primers specific to the target sequences. The final concentration of primers in qPCR mixture was 400 nM for mRNA detection and 200 nM for miRNA detection. To evaluate the expression levels of the selected genes, the SensiFast SYBR & Fluorescein Kit (Bioline Reagents Ltd., London, UK) was used.

The PCR reactions were carried out in a total volume of 10 μL, with the cDNA template accounting for no more than 10% of the total reaction volume. The reaction was performed using the CFX Connect Real-Time PCR Detection System (Bio-Rad Polska Sp. z o.o., Warsaw, Poland). No-reverse-transcription controls (NoRT) were included to assess potential genomic DNA contamination, whereas no-template controls (NTC) were used to verify the absence of reagent contamination and nonspecific amplification during qPCR.

The expression of the target gene was normalized to two reference genes: glyceraldehyde-3-phosphate dehydrogenase (*GAPDH*) and β-actin (*ACTB*). The *miRNA-27b-3p* levels were normalized to *snU6* expression levels. The primers characteristics are indicated in Table S1.

### 2.5. Protein Expression Analysis—Western Blot

To verify whether the phenotypic differences observed between the studied cell lines correlated with molecular changes, quantitative protein analysis was performed using Western blotting. For analysis, the cells were lysed using RIPA buffer (Sigma Aldrich/Merck, Poznan, Poland) with a 1% protease and phosphatase inhibitor cocktail (Thermo Fisher Scientific, Warsaw, Poland). Protein concentrations in each sample were determined using the Bicinchoninic Acid Assay kit (BCA; Thermo Fisher Scientific, Warsaw, Poland). The Laemmli buffer (4×; Bio-Rad, Hercules, CA, USA) was added to the prepared protein samples, and afterwards incubated in a T100 thermal cycler (Bio-Rad, Hercules, CA, USA) for 5 min at 95 °C.

Subsequently, proteins were separated by electrophoresis in a 12.5% polyacrylamide gel containing sodium dodecyl sulfate (SDS-PAGE; 100 V for 90 min). Electrophoresis was carried out in a Mini-PROTEAN Tetra vertical electrophoresis system (Bio-Rad, Hercules, CA, USA). After electrophoresis, proteins were transferred to polyvinylidene fluoride membranes (PVDF) in transfer buffer (Bio-Rad, Hercules, CA, USA) using a Mini Trans-Blot^®^ system (Bio-Rad, Hercules, CA, USA). The transfer was performed for 1 h at a constant voltage of 100 V.

Following the transfer, membranes were blocked for 1 h in 5% bovine serum albumin (BSA, Sigma Aldrich/Merck, Poznan, Poland) prepared in TBS-T (Tris-Buffered Saline with Tween 20, Bio-Rad, Hercules, CA, USA). To detect the proteins of interest, membranes were incubated overnight at 4 °C with primary antibodies. After incubation, membranes were washed five times with TBS-T and subsequently incubated with secondary antibodies for 1 h at 4 °C. After incubation, membranes were washed five times with TBS-T.

Chemiluminescent signal detection was performed using the Bio-Rad ChemiDoc™ XRS system (Bio-Rad, Hercules, CA, USA). The substrate used for signal development was DuoLuX^®^ Chemiluminescent and Fluorescent Peroxidase (HRP) substrate (Vector Laboratories, Biokom, Janki, Poland). The obtained results were analyzed using the Image Lab™ software (version 6.1; Bio-Rad: Hercules, CA, USA; 2020). The antibodies for protein detection used are indicated in Table S2.

### 2.6. Statistical Analysis

Results are shown as mean ± SD. Each experiment was conducted in two independent biological replicates, with every experimental condition measured in at least three technical replicates within each independent run. Given the structure of the dataset, statistical analysis was performed using mean values from independent biological replicates, in accordance with the approach routinely used by our group for this type of analysis [31]. The required sample size was determined according to Stein’s two-stage procedure, by which an initial set of independent replicates was used to estimate experimental variability, after which the number of replicates was adjusted to meet the minimum sample size required for statistical analysis [32].

Data distribution was assessed using the Shapiro–Wilk test, while equality of variances was evaluated with Levene’s test and, where appropriate, the Brown–Forsythe test. Group comparisons were performed using one-way analysis of variance (ANOVA) followed by Tukey’s post hoc test for multiple comparisons. In addition, Spearman’s rank correlation analysis was applied to assess the relationships between *miR-27b-3p* levels and IGF2BP3 expression at both the mRNA and protein levels. Correlations were considered relevant when r ≥ 0.5 or r ≤ −0.5, with *p* ≤ 0.05. All statistical analyses were carried out using GraphPad Prism version 10.6.1 (GraphPad Software, San Diego, CA, USA). Differences were considered statistically significant at *p* < 0.05.

## 3. Results

### 3.1. Primary Tumor- and Metastasis-Derived Canine Osteosarcoma Cell Lines Display Distinct Morphotypic Features In Vitro

**Figure 1:**
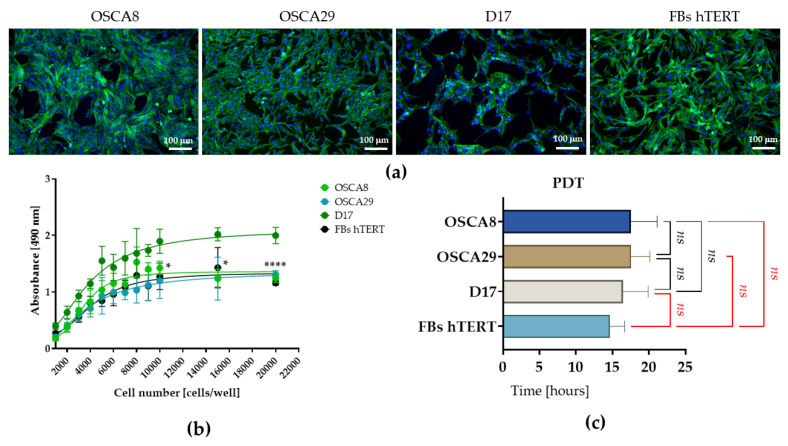
Morphology, metabolic activity, and doubling kinetics of canine osteosarcoma cell lines in vitro. Representative fluorescence images of OSCA8, OSCA29, D17, and hTERT fibroblasts at ~80% confluence revealed distinct morphologies and growth organization. OSCA8 and OSCA29 formed spindle-shaped adherent monolayers, whereas D17 cells showed a flattened epithelial-like phenotype with a branched network-like architecture. hTERT fibroblasts exhibited a typical fibroblast-like morphology (**a**). D17 cells showed the highest metabolic activity in the MTS assay, while OSCA-8, OSCA-29, and hTERT fibroblasts displayed comparable metabolic activity (**b**). In turn, PDT values were comparable across all analyzed cell lines (**c**). The data are presented as means ± SD. Statistically significant differences were indicated as follows: * *p*-value < 0.05 and **** *p*-value < 0.0001, while non-significant differences are denoted as *ns*.

Morphological evaluation performed at approximately 80% confluence revealed clear morphotypic differences among the analyzed cell lines (Figure 1a). OSCA8 and OSCA29 cells exhibited a spindle-shaped morphology and formed adherent monolayers, with a predominant mesenchymal, fibroblast-like phenotype. In contrast, D17 cells displayed a distinct epithelial-like appearance, characterized by flattened polygonal cells arranged in atypical, branched, interconnected networks with evident cell–cell contacts, rather than in a classic continuous and uniform monolayer. hTERT fibroblasts exhibited a typical fibroblast-like morphology, consisting of elongated, bipolar to spindle-shaped cells with a well-organized growth pattern, also forming an adherent monolayer.

Analysis of MTS absorbance as a function of cell number revealed clear inter-line differences in metabolic activity (Figure 1b). D17 cells consistently demonstrated the greatest metabolic activity across the tested seeding densities, whereas OSCA8, OSCA29, and hTERT fibroblasts showed broadly comparable metabolic profiles. However, this was not reflected in altered growth kinetics, as population doubling time (PDT) values remained comparable across all analyzed cell lines (Figure 1c).

Together, these findings suggest that primary tumor-derived OSCA cell lines and the metastasis-derived cell line D17 exhibit distinct growth architectures and metabolic profiles, despite a comparable proliferation rate (Figure 1).

### 3.2. Canine Osteosarcoma Cell Lines Exhibit Distinct G0/G1 and S-Phase Distributions

**Figure 2:**
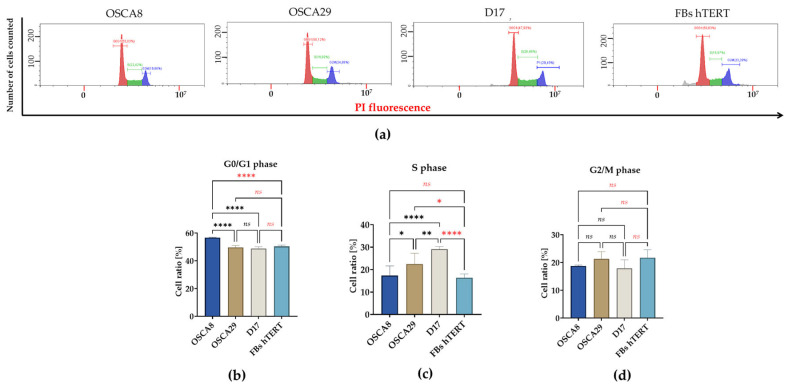
Cell cycle analysis of canine osteosarcoma cell lines and hTERT fibroblasts. Representative DNA content histograms illustrating the distribution of cells across cell cycle phases are shown in panel (**a**). Panels (**b**–**d**) present the quantitative analysis of the percentage of cells in the G0/G1, S, and G2/M phases, respectively. Data are presented as mean ± SD. Statistical significance was indicated as follows: * *p* < 0.05, ** *p* < 0.01, and **** *p* < 0.0001, while non-significant differences are denoted as *ns*.

Cell cycle analysis revealed significant differences in phase distribution among the analyzed cell lines (Figure 2). OSCA8 cells exhibited the highest proportion of cells in the G0/G1 phase, significantly exceeding those of OSCA29, D17, and hTERT fibroblasts, which showed comparable G0/G1 fractions (Figure 2a,b). Among the analyzed osteosarcoma cell lines, OSCA8 displayed the lowest proportion of S-phase cells, with values comparable to those observed in hTERT fibroblasts. OSCA29 exhibited an intermediate S-phase fraction, higher than OSCA8 but lower than D17, whereas D17 showed the highest proportion of cells undergoing DNA synthesis (Figure 2a,c). In contrast, the distribution of cells in the G2/M phase remained comparable across all analyzed lines (Figure 2a,d). Notably, these differences coexist with comparable population doubling times, indicating that cell cycle distribution alone does not directly translate into overall net growth kinetics under the applied culture conditions.

### 3.3. Canine Osteosarcoma Cell Lines Display Distinct Clonogenic and Migratory Phenotypes, with OSCA-8 Showing the Most Pronounced Aggressive Profile In Vitro

**Figure 3:**
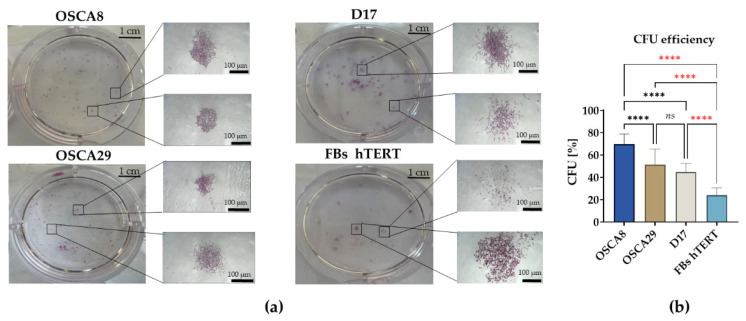
Clonogenic potential of canine osteosarcoma cell lines and hTERT fibroblasts. Representative images of culture wells after clonogenic assay and pararosaniline staining, together with magnified views of representative colonies acquired under a light microscope at 100-fold magnification (**a**); scale bars are indicated in the figure. Quantitative analysis of clonogenic potential expressed as CFU-E (%) in the analyzed cell lines (**b**). Data are presented as mean ± SD. Statistical significance was indicated as follows: **** *p* < 0.0001; non-significant differences are denoted as *ns*.

Clonogenic assay revealed clear differences in colony-forming capacity among the analyzed cell lines (Figure 3). OSCA8 displayed a pronounced ability to form colonies composed of more than 50 cells. Importantly, OSCA29 and D17 exhibited comparable clonogenic potential. Both OSCA8 and OSCA29 formed dense colonies, while D17 generated colonies of a more dispersed architecture. In contrast, hTERT fibroblasts exhibited limited colony-forming ability relative to the osteosarcoma lines, further highlighting the overall greater clonogenic capacity of the osteosarcoma cell lines (Figure 3a,b).

**Figure 4:**
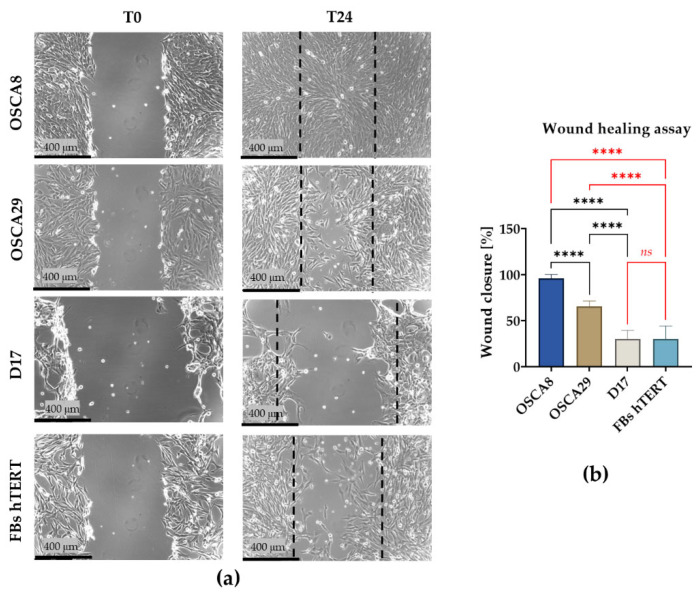
Migratory capacity of canine osteosarcoma cell lines and hTERT fibroblasts assessed by wound healing assay. Representative images showing the wound area at T0 and 24 h after scratching (**a**). Quantitative analysis of wound closure (%) in the analyzed cell lines (**b**). Data are presented as mean ± SD. Statistical significance was indicated by ****, representing *p* < 0.0001, while non-significant differences are denoted as *ns*. dashed lines indicate the wound edges at T0.

Wound healing assay demonstrated marked inter-line differences in migratory behavior (Figure 4). OSCA8 cells exhibited the highest wound closure percentage, indicating the strongest migratory potential among the analyzed cell lines. This finding was consistent with the high clonogenic potential observed in OSCA8 cells. OSCA29 showed an intermediate phenotype, with significantly lower migration than OSCA8 but higher wound closure than D17.

D17 cells displayed the lowest wound-closure capacity, which may, at least in part, be related to their distinct network-like architecture and tendency to grow as branched, interconnected cellular structures rather than forming a uniform monolayer. Notably, hTERT fibroblasts exhibited a wound closure rate comparable to that of D17 cells (Figure 4a,b).

**Figure 5:**
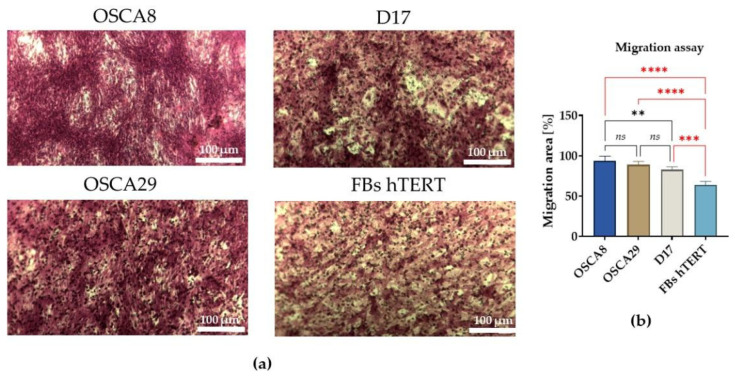
Comparative evaluation of transmembrane migratory capacity in canine osteosarcoma cell lines and hTERT fibroblasts. Representative images of cells that migrated through the membrane in the transwell migration assay. Scale bars are indicated in the images (**a**). Statistical analysis of migratory activity, expressed as migration area (%), in the analyzed cell lines (**b**). Data are presented as mean ± SD. Statistical significance was indicated as follows: ** *p* < 0.01, *** *p* < 0.001, and **** *p* < 0.0001; non-significant differences are denoted as *ns*.

Analysis of transmembrane migratory ability confirmed the prominent migratory potential of the OSCA8 osteosarcoma cell line (Figure 5). Nevertheless, this difference reached statistical significance only in comparison with D17 cells, whereas OSCA8 and OSCA29 showed comparable migration levels. Importantly, all osteosarcoma cell lines exhibited greater migratory activity than hTERT fibroblasts, in line with their tumor-derived origin and the expected biological differences between neoplastic and non-neoplastic cells (Figure 5a,b).

### 3.4. Distinct Post-Transcriptional Patterns of *IGF2BP3* Expression Are Observed Across Canine Osteosarcoma Cell Lines Despite Partially Concordant miR-27b-3p and Transcript Profiles

**Figure 6:**
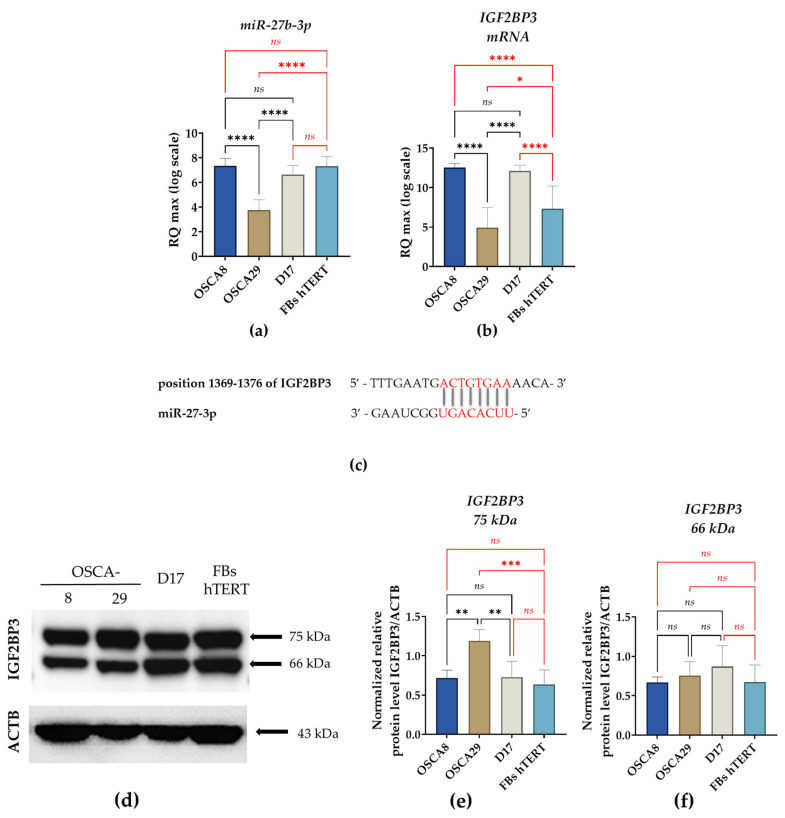
Comparative analysis of *miR-27b-3p* and IGF2BP3 expression in canine osteosarcoma cell lines. The analysis included the evaluation of relative *miR-27b-3p* expression (**a**) and *IGF2BP3* mRNA levels (**b**). In addition, the predicted binding site of *miR-27b-3p* within the IGF2BP3 transcript, including the seed region, is shown in panel (**c**). Intracellular *IGF2BP3* accumulation was assessed by Western blotting; representative blots are presented in panel (**d**), while panels (**e**,**f**) show the quantitative densitometric analysis of the identified IGF2BP3 immunoreactive bands. The results are shown as mean ± SD. Statistical significance was defined as * *p*-value < 0.05; ** *p*-value < 0.01; *** indicates *p* < 0.001, and **** indicates *p* < 0.0001, while non-significant differences are denoted as *ns*.

Analysis of *miR-27b-3p* expression showed that both OSCA8 and D17 exhibited higher levels of this miRNA, with values comparable to those observed in hTERT fibroblasts. In contrast, OSCA-29 had significantly lower *miR-27b-3p* levels compared with the other analyzed cell lines (Figure 6a).

A similar expression pattern was observed for IGF2BP3 mRNA (Figure 6b). OSCA8 and D17 were characterized by increased transcript levels, whereas OSCA29 showed significantly reduced IGF2BP3 mRNA expression. In hTERT fibroblasts, IGF2BP3 mRNA expression was lower than in OSCA8 and D17, but higher than in OSCA29 (Figure 6b). Furthermore, a potential interaction between *miR-27b-3p* and IGF2BP3 mRNA was identified (Figure 6c).

Western blot analysis detected two bands corresponding to approximately 75 kDa and 66 kDa (Figure 6d). Densitometric analysis demonstrated that the abundance of the 66 kDa band did not differ significantly among the examined cell lines. In contrast, the intensity of the 75 kDa band was significantly higher in OSCA29 cells than in the remaining cell lines (Figure 6d–f).

**Figure 7:**
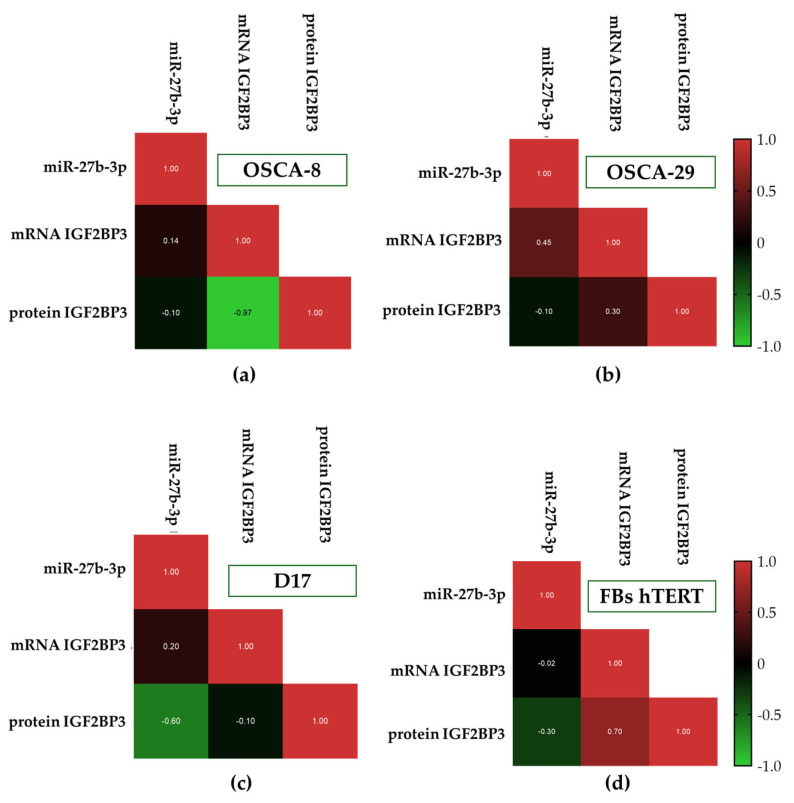
Triangular heatmaps showing Spearman’s rank correlation coefficients (ρ) for the relationships between *miR-27b-3p* expression and IGF2BP3 levels. The analysis was performed for OSCA8 (**a**), OSCA29 (**b**), D17 (**c**), and canine hTERT fibroblasts (**d**). Color intensity reflects the magnitude of the correlation, with blue indicating positive correlations and green indicating negative correlations.

Correlation analysis did not reveal significant association between *miR-27b-3p* and IGF2BP3 expression at either the transcript or protein level in the analyzed cell lines (Figure 7a–d). The only statistically significant finding was observed in OSCA8 cells, where IGF2BP3 mRNA and protein levels showed a strong inverse correlation (Spearman’s r = −0.97, *p* = 0.0333; Figure 7a). By contrast, the remaining models (OSCA29, D17, and hTERT) showed only non-significant, cell line-dependent directional tendencies (Figure 7b,c, respectively). These included a positive tendency between *miR-27b-3p* and IGF2BP3 mRNA in OSCA29 cells, a moderate inverse tendency between *miR-27b-3p* and IGF2BP3 protein in D17 cells, and a positive tendency between IGF2BP3 transcript and protein levels in hTERT fibroblasts. As these associations did not reach statistical significance, they should be considered exploratory.

## 4. Discussion

Osteosarcoma is an aggressive primary malignant bone tumor characterized by high biological heterogeneity, making it a major challenge not only in clinical management but also in preclinical research. This complexity affects both human experimental systems and canine-based models, as tumor phenotypic diversity may substantially influence cellular behavior in vitro and responses to therapeutic interventions [1,11,12].

In canine osteosarcoma, such heterogeneity should be regarded not only as a feature relevant to comparative oncology but also as an inherent property of the disease itself [1,12,17]. Accordingly, the distinct biological behavior observed among canine OSA cell lines further highlights the need for multiple complementary in vitro models to more fully capture the complexity of osteosarcoma biology [10,11].

Megquier et al. showed that several canine osteosarcoma cell lines, including OSCA8, retain key genomic features of primary canine OSA tissues, underscoring their relevance as in vitro models for preclinical investigation [33]. Consequently, the use of well-characterized canine OSA cell lines remains essential for both mechanistic research and the development of more precisely tailored experimental approaches [12,15]. At the same time, it should be noted that not all canine osteosarcoma cell lines used in basic research have been equally well characterized, which complicates data interpretation and cross-study comparisons [10,15,33,34].

In this study, we characterized three canine osteosarcoma cell lines—OSCA8, OSCA29, and D17. Although the use of three cell lines does not capture the full biological heterogeneity of canine osteosarcoma, the combined functional and molecular profiling performed here provides valuable reference data, particularly for the less extensively characterized OSCA29 model.

The cytophysiological behavior of canine osteosarcoma cells was assessed relative to non-neoplastic canine hTERT fibroblasts. The analysis included assessment of cell morphology, metabolic activity, proliferation, clonogenicity, and migratory capacity, as well as the expression of molecules potentially involved in the regulation of the aggressive phenotype, namely IGF2BP3 and *miR-27b-3p*, thereby capturing the distinct profiles of the investigated in vitro models.

Both OSCA8 and D17 are well-recognized canine osteosarcoma cell lines widely used in in vitro research as representative models of canine OSA reflecting their relevance for investigating biologically and clinically important aspects of canine osteosarcoma, including tumor cell behavior, treatment susceptibility, and regulatory pathway dysregulation [16,35,36,37,38]. In contrast, OSCA29 remains less extensively characterized and less commonly discussed in the literature, despite its potential value as an additional canine OSA model [10].

The morphology of the analyzed canine osteosarcoma cell lines was generally consistent with the mesenchymal origin of this tumor [11,36]. OSCA8 and OSCA29, both established from primary tumor tissue [10], exhibited a pronounced spindle-shaped phenotype and formed distinct, relatively homogeneous monolayers. By contrast, D17 showed a more heterogeneous morphology, with noticeable epithelial-like features and a more complex, network-like cellular architecture rather than the regular monolayer pattern observed in the primary tumor-derived lines. The consistency of these morphological observations with previous reports by Gebhard et al. for D17 [18] and Małek et al. for OSCA8 [35] supports the reproducibility of these models, while also underscoring the biological relevance of morphological assessment as an indicator not only of cytoskeletal organization, but also of unique growth architecture, intercellular organization, and line-specific patterns of colony formation and expansion in vitro.

Whereas the characteristic morphology of D17 and OSCA8 has been documented in earlier studies, detailed morphological characterization of OSCA29 has, to our knowledge, not yet been reported. This makes the present characterization of OSCA29 a valuable addition to the existing knowledge of canine osteosarcoma in vitro models.

In the present study, we have analyzed the proliferative activity using complementary assays, including an indirect indicator of viable cell metabolic activity and growth (MTS), as well as evaluation of population doubling time, cell cycle analysis, and clonogenic growth. The obtained results clearly indicate that proliferation in canine OSA models cannot be reduced to a single parameter, such as cell cycle distribution alone, especially since DNA-content-based cell cycle analysis may be complicated by the high aneuploidy and copy number variability characteristic of OSA tumors [39].

We showed that D17 cells displayed the highest metabolic activity and the greatest proportion of S-phase cells, consistent with a highly active metabolic and proliferative state. Notably, population doubling time remained comparable between osteosarcoma lines and hTERT canine fibroblasts, indicating that, under the applied in vitro conditions, these cells achieved a broadly similar rate of expansion. The PDT value is shaped not only by the intrinsic proliferative state of the cells, but also by their viability and adaptation to culture conditions [40]. Thus, the efficiency with which growth-related processes are translated into overall population increase does not seem to directly correspond to the progression-related potential of these cell lines, as evidenced by the significantly higher clonogenic and migratory behaviors observed in the osteosarcoma models compared with canine hTERT fibroblasts.

Among the analyzed osteosarcoma cell lines, OSCA8 exhibited the greatest clonogenic capacity together with the highest migratory activity in the wound-healing assay.

The higher clonogenic potential of OSCA8 relative to D17 observed in the present study is partially consistent with the findings of Musser et al., who reported higher normalized clonogenic survival of OSCA8 compared with D17 following ascorbate treatment [41]. Although their results refer to relative post-treatment survival rather than basal clonogenicity per se, both sets of data point in the same direction and may indicate a greater long-term capacity of OSCA8 cells to maintain colony formation.

Interestingly, the transwell assay revealed broadly comparable transmembrane migratory activity among the analyzed canine osteosarcoma cell lines. Although both OSCA8 and D17 have been regarded as aggressive canine OSA models [10,18,19,35], the present results suggest that they reflect different functional manifestations of tumor aggressiveness. D17 was distinguished primarily by a proliferatively engaged phenotype, whereas OSCA-8 exhibited the strongest clonogenic capacity and the highest migratory activity in the wound-healing assay. By comparison, OSCA29 was not characterized in the available literature with respect to these functional features; nevertheless, our findings support its classification as an intermediate model [10], positioned between the more distinct phenotypes represented by D17 and OSCA8.

To complement the cytofunctional assessment of the analyzed canine osteosarcoma cell lines, we further investigated the expression of *miR-27b-3p* and IGF2BP3 at the transcript and protein levels. Both molecules have been implicated in osteosarcoma biology and are regarded as molecular factors potentially associated with tumor progression and aggressive phenotype-related regulation [23,28,42,43]. Moreover, the significance of IGF2BP3 in bone tumor biology extends beyond osteosarcoma. In Ewing sarcoma, IGF2BP3 has been associated with inherited susceptibility, prognosis, and the modulation of phenotypic heterogeneity, highlighting its broader relevance as a regulator of tumor progression in primary bone malignancies [22,44,45]. Notably, available data on their expression and functional relevance derive from human osteosarcoma models, while analogous evidence in canine OSA remains scarce. Therefore, our findings could fill this gap by providing a preliminary molecular context for the phenotypic diversity observed across the investigated canine cell lines.

Consistent with previous observations in human osteosarcoma, miR-27-3p has been proposed as a pro-proliferative miRNA that promotes the G1/S transition and enhances tumor cell growth [28]. In our study, this relationship was only partially confirmed. The D17 cells with relatively high *miR-27b-3p* expression had the highest S-phase fraction, suggesting a role for this miRNA in cell-cycle progression. However, OSCA8 cells also expressed high levels of *miR-27b-3p* while exhibiting the lowest proportion of S-phase cells, indicating that this relationship is not uniform across canine OSA models. Therefore, the expression of *miR-27b-3p* and its correlation with proliferative behavior in canine osteosarcoma is likely context-dependent.

The relatively high expression of *miR-27b-3p* observed in OSCA8 and D17 could be broadly consistent with their more aggressive functional profiles. In contrast, OSCA29 exhibited the lowest expression of both *miR-27b-3p* and IGF2BP3 mRNA, which may reflect its more moderate phenotype with respect to the analyzed functional parameters. However, this pattern was not mirrored at the protein level.

Although *miR-27b-3p* was predicted to target IGF2BP3, including within the seed region, no consistent linear association between *miR-27b-3p* and IGF2BP3 mRNA levels was observed across the analyzed canine osteosarcoma cell lines. To our knowledge, this is the first analysis to address this potential regulatory relationship in canine osteosarcoma models, suggesting that it may be influenced by additional cell-context-dependent mechanisms and requiring further functional validation.

Further, blotting analysis to detect IGF2BP3 protein revealed two IGF2BP3-immunoreactive bands, potentially corresponding to two molecular forms. Of these, the 66 kDa band remained broadly comparable across all analyzed cell lines, including hTERT fibroblasts, whereas the 75 kDa band showed the highest accumulation in OSCA29. These findings indicate that transcript abundance was not directly reflected in the protein pattern, suggesting that IGF2BP3 regulation in canine OSA cells may involve additional post-transcriptional or post-translational mechanisms. A similar lack of direct correspondence between IGF2BP3 mRNA and protein expression has been reported in melanoma, where IGF2BP3 transcript levels correlated more closely with clinicopathological features than the corresponding protein expression, supporting the view that mRNA and protein levels may provide partially distinct biological information [46].

This interpretation of the IGF2BP3-*miR-27b-3p* axis is further supported by the correlation analysis, which did not reveal a consistent association between *miR-27b-3p* and IGF2BP3 expression across the analyzed models. The only statistically significant relationship was observed in OSCA8, where IGF2BP3 mRNA and protein levels showed a strong inverse correlation, suggesting marked transcript–protein dissociation in this cell line. In the remaining models, only non-significant, line-specific tendencies were observed, including a positive *miR-27b-3p*/*IGF2BP3* mRNA trend in OSCA29, a negative *miR-27b-3p*/IGF2BP3 protein trend in D17, and a positive mRNA–protein tendency in hTERT fibroblasts.

Collectively, the obtained findings indicate that the molecular background of the analyzed canine OSA models is context-dependent and cannot be reduced to a simple linear relationship between *miR-27b-3p* expression, *IGF2BP3* transcript abundance, and IGF2BP3 protein accumulation. Although the basal expression profile of these markers, characterized among the studied cell lines, does not appear to provide a direct molecular explanation for the functional differences observed, it nevertheless adds an important dimension to the overall characterization of canine osteosarcoma cell lines. Rather than defining a uniform regulatory axis, the obtained expression patterns support the view that phenotypic heterogeneity in canine osteosarcoma is linked to more complex, cell-line-specific regulatory mechanisms [12]. Indeed, our findings should be considered as hypothesis-generating, but may provide a rationale for future studies combining transcriptomic profiling with functional *miR-27b-3p* modulation and IGF2BP3 mRNA/protein assessment to determine whether *miR-27b-3p* directly contributes to IGF2BP3 regulation in canine osteosarcoma cells.

An important aspect of the present study is the direct comparative characterization of OSCA29 alongside the more widely used OSCA8 and D17 models. In contrast to these better-established models, OSCA29 remains relatively poorly described in the available literature. We have noted a more moderate functional profile of OSCA29, further distinguished by low *miR-27b-3p* and *IGF2BP3* mRNA expression together with a distinct IGF2BP3 protein pattern. These findings indicate that OSCA29 may represent a unique and biologically relevant model that expands the currently available spectrum of canine OSA in vitro systems [10,17]. The observed intermediate phenotype of OSCA29 may also reflect unique genetic or epigenetic features acquired during tumor evolution or adaptation to in vitro culture conditions—a phenomenon well-documented across multiple osteosarcoma models [11,47].

Moreover, the analysis of hTERT canine fibroblasts provided a useful non-neoplastic reference for comparative purposes; however, the immortalized nature of this line should be taken into account when interpreting proliferation-related parameters, which reflected overall in vitro growth potential rather than tumor-specific proliferative behavior alone. Nevertheless, the osteosarcoma cell lines remained clearly distinguishable from hTERT fibroblasts in terms of clonogenic and migratory activity, supporting the malignant-specific functional properties of the OSA models. The noted expression of IGF2BP3 in hTERT cells may also reflect immortalization-associated features rather than malignant transformation per se. This view is supported by recent data showing that IGF2BP3 may also function as a broader regulator of metabolically active and proliferatively competent cell states, including non-malignant stem/progenitor populations [43]. To our knowledge, this is the first study to characterize this canine hTERT fibroblast line in the context of comparative osteosarcoma research, although future comparisons with primary canine fibroblasts will be needed to clarify whether IGF2BP3 expression is model-specific or reflects a broader feature of proliferative stromal cells.

## 5. Conclusions

Taken together, the present results show that canine osteosarcoma cell lines are not equivalent biological models, and their selection should be carefully considered and guided by the specific functional and molecular characteristics most relevant to the experimental objective. Future studies including broader panels of canine osteosarcoma cell lines and primary tumor-derived samples would be warranted to further validate these findings and better reflect intertumoral heterogeneity. Moreover, although no therapeutic intervention was included at this stage, the generated baseline dataset is intended to support the rational design and interpretation of future drug- and radiation-response studies.

A limitation of the study is that the molecular analyses were exploratory and did not include functional experiments to directly verify the regulatory relationship between *miR-27b-3p* and IGF2BP3. Despite these limitations, the study provides a relevant comparative framework for canine OSA in vitro models and constitutes an initial step toward clarifying the potential role of IGF2BP3-associated regulation in the biological heterogeneity of this tumor.

## Data Availability

The raw data supporting the conclusions of this article will be made available by the authors on request. The raw data are openly available in the Repository of the Wrocław University of Environmental and Life Sciences at https://bazawiedzy.upwr.edu.pl/info/researchdata/UPWR402f1660034c411d8ef1c882e763c103/ (accessed on 14 April 2026) for the original microscopic data and at https://bazawiedzy.upwr.edu.pl/info/researchdata/UPWRafc1ea55f9914daaa9caa76035fdf90a/ (accessed on 14 April 2026) for the Western blot data. In addition, the data supporting this study are available within the article and its Supplementary Materials.

## Supplementary Materials

The following supporting information can be downloaded at: https://www.mdpi.com/article/10.3390/cells15100878/s1; Table S1:Detailed characteristics of oligonucleotide primers used for RT-qPCR analysis; Table S2: List of antibodies used for Western blot analysis, including the applied working dilutions.

## Abbreviations

The following abbreviations are used in this manuscript:

*ACTB*	β-actin
AKT	Protein Kinase B
BCA	Bicinchoninic Acid Assay
BSA	Bovine Serum Albumin
CFU	Colony-Forming Unit
CGM	Complete Growth Medium
DAPI	4′,6-diamidino-2-phenylindole dihydrochloride
DMEM	Dulbecco’s Modified Eagle Medium
EMEM	Eagle’s Minimum Essential Medium
FBS	Fetal Bovine Serum
*GAPDH*	Glyceraldehyde-3-Phosphate Dehydrogenase
HBSS	Hanks’ Balanced Salt Solution
HRP	Chemiluminescent and Fluorescent Peroxidase
IGF-1R	Insulin-like Growth Factor 1 Receptor
IGF2BP3	Insulin-like Growth Factor 2 mRNA-Binding Protein 3
IGF-I/GH	Insulin-like growth factor 1/Growth Hormone
MAPK	Mitogen-Activated Protein Kinases
OSA	Osteosarcoma
P/S	Penicillin-Streptomycin
PFA	paraformaldehyde
PVDF	Polyvinylidene Fluoride
TBS	Tris-Buffered Saline with Tween 20

## Appendix A

**Table A1:** Origin and basic characteristics of the canine osteosarcoma cell lines used in the study.

Parameter/Cell Line	OSCA-8	OSCA-29	D17
Morphology	spindle-shaped	spindle-shaped	epithelial-like
Tissue of origin	Bone	bone	bone
Tumor origin	primary tumor	primary tumor	lung metastasis
Age of donor dog (years)	1	6	11
Breed	Rottweiler	Rottweiler	Poodle
Sex	Male	Female	Female
